# First Therapeutic Approval for Eosinophilic Esophagitis

**DOI:** 10.3390/gastroent13030024

**Published:** 2022-07-30

**Authors:** Rami A. Al-Horani, Raquel Chiles

**Affiliations:** Division of Basic Pharmaceutical Sciences, College of Pharmacy, Xavier University of Louisiana, New Orleans, LA 70125, USA

**Keywords:** esophagitis, inflammation dupilumab, monoclonal antibodies

## Abstract

Eosinophilic esophagitis (EE) is a chronic, immune-mediated or antigen-mediated esophageal disease. Treatment for patients with EE can be challenging with no previously approved medications. Current management strategies follow the four D’s paradigm of drugs, dietary elimination, dilation, and disease anxiety and hypervigilance therapy. On 20 May 2022, dupilumab was approved by FDA for EE. A dose of 300 mg dupilumab weekly significantly improved signs and symptoms of EE compared to placebo in a phase 3 trial. The approval of dupilumab will fulfill an unmet need for the increasing number of patients with EE.

## 1. Introduction

### 1.1. Definition and Symptoms

Eosinophilic esophagitis (EE) is a chronic, immune-mediated or antigen-mediated esophageal disease. In EE, large numbers of eosinophils are found in the inner lining of the esophagus [1,2]. The chronic inflammation of EE leads to several symptoms. EE is characterized by symptoms relevant to eosinophil-predominant inflammation and esophageal dysfunction. The prevailing antigens that trigger EE seem to be food related. Other causes of EE include allergic vasculitis, parasitic infection, esophageal leiomyomatosis, and Crohn’s disease of the esophagus [3]. Symptoms of EE include vomiting, feeding problems, and dysphagia and food impaction in adults and adolescents and abdominal pain in children. Present also is esophageal mucosal eosinophilia of ≥15 eosinophils per high-power field [1,2].

### 1.2. Pathogenesis

Premature delivery, birth by caesarean, lack of breast-feeding, antibiotic exposure during infancy, food allergy, and living in low population density areas have all been associated with EE. Studies have also indicated that the absence of early exposure to microbes or the altered microbiome in vulnerable patients may play a role [4–6]. The male predominance of EE and studies of genome-wide association and family history indicate that there is a genetic component to EE. In fact, three genes encoding thymic stromal lymphopoietin, eotaxin-3, and calpain-14 are altered in EE [7–12]. Furthermore, dilated interepithelial spaces, altered function of epithelial barrier, and down-regulation of filaggrin, zonulin-1, and desmoglein-1 have been found upon assessing esophageal tissue of EE patients. Altered epithelial permeability leads to an uncontrolled environment, enhances antigen presentation, and results in recruitment of eosinophils [13–17].

Several lines of evidence also suggest that EE can be mediated by type 2 helper T (Th2) cell activity and promoted largely by food antigens [3]. Specifically, interleukin-5 and interleukin-13 may play a role in the pathogenesis of EE [18]. There are also thymic stromal lymphopoietin-directed and cytokine-directed proliferation and recruitment of eosinophils, IgE-bearing mast cells, Th2 lymphocytes, natural killer cells, and basophils in EE patients [19–22]. Interestingly, food-specific IgG4 was shown to be present in the esophageal epithelium and to be reactive to the four most common food-antigen triggers in EE patients [23].

### 1.3. Prevalence

EE has been described in all age groups. Nevertheless, it mainly impacts white men with an onset from school age to midlife [24]. A history of atopic disorders, such as eczema, rhinitis, asthma, and anaphylactic food allergy is widespread among patients predisposed to EE [25]. Patients undergoing endoscopy owing to food impaction history have a significantly higher incidence of up to 54% [26]. Studies have estimated the prevalence of EE as 1–5 per 10,000 people in the western hemisphere, with increased prevalence in Asia [27–30].

### 1.4. Complications

EE is a long-lasting inflammatory condition with a persistent course of instability. Life expectancy does not appear to be affected, but EE often affects the life quality. Furthermore, there appear no association between EE and esophageal cancer; nevertheless, there are serious concerns that the uncontrolled, prolonged inflammation may lead to irreversible structural changes resulting in stricture formation, tissue fibrosis, and impairment of the esophageal function. Other complications of EE include esophageal perforation, food impaction, malnutrition, hepatic portal venous gas, achalasia-like changes, and adrenal insufficiency. Concurrent conditions that have been associated with patients with EE include connective tissue diseases [31], Crohn’s disease [32], and celiac disease [33].

### 1.5. Current Treatment Strategies

**Figure 1.**
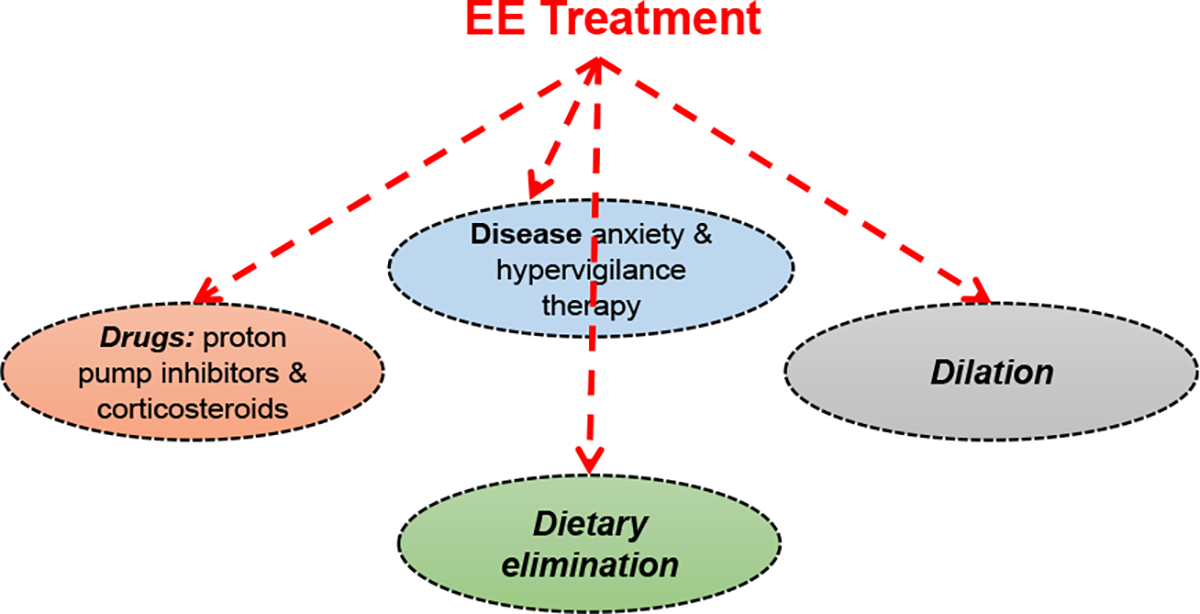
The current paradigm of EE treatment. The treatment follows the 4 Ds concept.

Some adults and many children with EE show improvement with proton pump inhibitors [34–36] as well as diet modification so that the food that triggers allergy is removed, particularly egg, milk, wheat, soy, nuts, and fish [37,38]. Some patients require a liquid formula diet provided via a feeding tube. Steroid medications can be used to control inflammation [39,40]. Esophageal dilation to relieve esophageal narrowing is also an established strategy for EE, especially in older adults and teenagers [41]. Generally, the current paradigm of EE treatment is depicted in Figure 1. The paradigm revolves around the four Ds concept of drugs (proton pump inhibitors or corticosteroids), dietary elimination, dilation, and disease anxiety and hypervigilance therapy.

## 2. Dupilumab (Dupixent^®^, Regeneron)

The drug was jointly developed by Sanofi Genzyme and Regeneron Pharmaceuticals. Dupilumab has an approximate molecular weight of 147 kDa. Dupilumab is produced by recombinant DNA technology in Chinese Hamster Ovary cell suspension culture. Dupilumab is a human monoclonal IgG4 antibody that inhibits both interleukin-13 (IL-13) and interleukin-4 (IL-4) signaling by binding to the IL-4Rα subunit. Such binding inhibits IL-13 and IL-4 cytokine-induced inflammatory responses, including the release of proinflammatory cytokines, chemokines, immunoglobulin E, and nitric oxide [42,43]. It was previously approved for moderate to severe eosinophilic or oral glucocorticoid-dependent asthma, moderate to severe atopic dermatitis, and chronic rhinosinusitis with nasal polyposis [44].

Following subcutaneous administration, its bioavailability is 61% to 64%. Its time to peak is ~1 week. Its Vd is ~4.8 ± 1.3 L. Monoclonal antibodies are primarily cleaved to short peptides and individual amino acids by catabolism. The median times to reach nondetectable levels are 10 to 11 weeks (for 300 mg every 2 weeks), 13 weeks (for 300 mg weekly), and 9 weeks (for 200 mg every 2 weeks) [45,46].

On 20 May 2022, the indication for dupilumab was expanded to include treating EE in people aged 12 years or older and weighing at least 40 kg. The efficacy and safety of dupilumab in EE was exhibited in a randomized, double-blind, parallel-group, multicenter, placebo-controlled trial that included two 24-week treatment periods (Parts A and B, phase 3, *n* = 321) that were independently conducted in separate groups of patients. In both Parts A and B, patients received 300 mg dupilumab or placebo every week. The primary outcome measures were the proportion of patients achieving peak esophageal intraepithelial eosinophil count of ≤6 eosinophils per high-power field at week 24 and the absolute change in Dysphagia Symptom Questionnaire (DSQ) score. In Part A, 60% of the patients who received dupilumab achieved the pre-determined level of decrease of eosinophils in the esophagus in relative to only 5% of those in the placebo group. Patients who received dupilumab also had an average improvement of 22 points in the DSQ score relative to only 10 points for patients in the placebo group. In Part B, 59% of patients who were treated with dupilumab attained the pre-determined level of decrease of eosinophils in the esophagus relative to 6% of those in the placebo group. Overall, in this phase 3 trial, dupilumab 300 mg weekly substantially alleviated signs and symptoms of EE compared to placebo [47].

There are no dosage adjustments provided in the manufacturer’s labeling for patients with altered kidney or liver functions. Dupilumab can cause allergic reactions, keratitis, and conjunctivitis because of its immunosuppressive effects. It can also reactivate cold sores. Patients who had been treated with dupilumab had decreased levels of T helper cells [42–44]. No animal studies have been performed to evaluate the mutagenic or carcinogenic potential of dupilumab. No effects on fertility parameters such as menstrual cycle length, reproductive organs, or sperm analysis were detected in sexually mature mice that were injected with a homologous antibody against IL-4Rα at doses up to 200 mg/kg/week.

## 3. Discussion of the Study

The FDA approval was based on results of a phase 3 trial with two parts evaluating the efficacy and safety of Dupixent 300 mg weekly compared with placebo. A single randomized, double-blind, multicenter, parallel-group, placebo-controlled trial, including two 24-week treatment periods (Parts A and B), was conducted in adult and pediatric subjects 12 to 17 years of age, weighing at least 40 kg, with EE (NCT03633617). In the two parts, subjects were randomized to receive 300 mg dupilumab every week or placebo. Eligible subjects had ≥15 intraepithelial eosinophils per high-power field following a treatment course of a proton pump inhibitor either prior to or during the screening period and symptoms of dysphagia as measured by the DSQ. At baseline, 43% of subjects in Part A and 37% of subjects in Part B had a history of prior esophageal dilations.

Baseline characteristics and demographics were similar in the two parts. A total of *n* = 81 subjects (61 adults and 20 pediatric subjects) were enrolled in Part A and *n* = 159 subjects (107 adults and 52 pediatric subjects) were enrolled in Part B. The mean age in years was 32 years (range of 13–62 years) in Part A and 28 years (range of 12–66 years) in Part B. Most of subjects were male (60% in Part A and 68% in Part B) and white (96% in Part A and 90% in Part B). The mean baseline DSQ score (standard deviation) was 33.6 (12.4) in Part A and 37.2 (10.7) in Part B.

As mentioned above, the primary efficacy endpoints were the proportion of subjects achieving histological remission defined as peak esophageal intraepithelial eosinophil count of ≤6 eosinophils per high-power field at week 24 and the absolute change in the subject-reported DSQ score from baseline to week 24. Efficacy results are reported in Table 1 [48].

In the two parts, a larger proportion of participants randomized to dupilumab achieved histological remission compared to placebo. At week 24, treatment with the drug also led to a significant improvement in LS mean change in DSQ score compared to placebo. The results of the anchor-based analyses that incorporated the participants’ perspectives suggested that the observed improvement in dysphagia from the two parts is representative of a clinically meaningful within-subject improvement.

In the above trial, the proportion of participants who discontinued treatment due to adverse events was 2% of the placebo group and 2% of the dupilumab 300 mg QW group.

Table 2 summarizes the adverse reactions that occurred at a rate of at least 2% in participants treated with dupilumab and at a higher rate than in their respective comparator group in the two parts.

Generally speaking, the safety profile of dupilumab in 72 pediatric subjects 12 to 17 years of age, weighing at least 40 kg, and adults in Parts A and B was similar.

It is important to mention here that a phase 2 trial of adults with active EE (two episodes of dysphagia per week with peak esophageal eosinophil density of ≥15 eosinophils per high-power field) was conducted earlier from 12 May 2015 through 9 November 2016, at multiple sites (NCT02379052) [49]. For 12 weeks, the adults were randomly assigned to two groups: the first group was the patients who received weekly subcutaneous injections of dupilumab (300 mg, *n* = 23) and the second group was the placebo group (*n* = 24). The primary endpoint was changed from baseline to week 10 in Straumann Dysphagia Instrument patient-reported outcome score. At the conclusion of the study, it was found that dupilumab significantly decreased dysphagia, histologic features of disease (including eosinophilic infiltration and a marker of type 2 inflammation), and abnormal endoscopic features compared with placebo. Dupilumab also significantly increased esophageal distensibility and was generally well-tolerated [49].

## 4. Conclusions

There are about 160,000 patients in the US living with EE who are currently using therapeutics not necessarily approved for the disease. Among them, about 48,000 continue to experience EE symptoms regardless of several treatments. The FDA granted dupilumab priority review and breakthrough therapy designations for EE, and on 20 May 2022 it was approved by the FDA for EE. Treatment for patients with EE can be challenging with no previously approved medications. Patients now have a treatment available to control their symptoms, improve inflammation, and heal the changes in the esophagus. Importantly, the approval of this drug for EE underscores the role of type 2 inflammation in this disease [47,49,50]. The recommended dosage of dupilumab for adult and pediatric patients 12 years of age and older, weighing at least 40 kg, is 300 mg given every week (QW).

## Figures and Tables

**Table 1.** Efficacy results of dupilumab at week 24 in subjects 12 years of age and older with EE.

	Part A			Part B		
	
	Dupilumab 300 mg QW ^c^ *n* = 42	Placebo ^c^ *n* = 39	Difference vs. Placebo (95% CI) ^c^	Dupilumab 300 mg QW ^c^ *n* = 80	Placebo ^c^ *n* = 79	Difference vs. Placebo (95% CI) ^c^

**Primary Endpoints**						

**Proportion of subjects achieving histological remission (peak esophageal intraepithelial eosinophil count ≤6 eos/hpf ^a^), *n* (%)**	25 (59.5)	2 (5.1)	57.0 (40.9, 73.1)	47 (58.8)	5 (6.3)	53.5 (41.2, 65.8)

**Absolute change from baseline in DSQ score (0–84 ^b^), LS mean (SE)**	−21.9 (2.5)	−9.6 (2.8)	−12.3 (−19.1, −5.5)	−23.8 (1.9)	−13.9 (1.9)	−9.9 (−14.8, −5.0)

a Eosinophils per high-power field.

b Total biweekly DSQ scores range from 0 to 84; higher scores indicate greater frequency and severity of dysphagia.

c For histological remission, the difference in percentages is estimated using the Cochran–Mantel–Haenszel method, adjusting for randomization stratification factors. For absolute change in DSQ score, the LS mean changes, standard errors, and differences are estimated using an ANCOVA model with treatment group, randomization stratification factors, and baseline measurement as covariates.

**Table 2.** Adverse reactions occurring in ≥2% of patients with EE treated with dupilumab in a placebo-controlled trial (Parts A and B; 24-week safety pool).

	Parts A and B	
Adverse Reaction	Dupilumab 300 mg QW *n* = 122 *n* (%)	Placebo *n* = 117 *n* (%)
**Injection site reaction** ^a^	46 (38%)	39 (33%)
**Upper respiratory tract infection** ^b^	22 (18%)	12 (10%)
**Arthralgia**	3 (2%)	1 (1%)
**Herpes viral infections** ^c^	3 (2%)	1 (1%)

a Injection site reactions are composed of several terms including, but not limited to, injection site swelling, pain, and bruising.

b Upper respiratory tract infections are composed of several terms including, but not limited to, COVID-19, sinusitis, and upper respiratory tract infection.

c Herpes viral infections are composed of oral herpes and herpes simplex.

## Data Availability

Provided in the manuscript.
